# Microstructure and Corrosion Resistance of 7075 Aluminium Alloy Composite Material Obtained from Chips in the High-Energy Ball Milling Process

**DOI:** 10.3390/ma17215331

**Published:** 2024-10-31

**Authors:** Barbara Kościelniak, Diana Groch, Wojciech J. Nowak, Marcin Drajewicz, Przemysław Kwolek

**Affiliations:** Department of Materials Science, Faculty of Mechanical Engineering and Aeronautics, Rzeszow University of Technology, al. Powstańców Warszawy 12, 35-959 Rzeszow, Poland; b.koscielnia@prz.edu.pl (B.K.); dgroch97@gmail.com (D.G.); wjnowak@prz.edu.pl (W.J.N.); drajewic@prz.edu.pl (M.D.)

**Keywords:** high-energy ball milling, aluminium alloy chips, microstructure, corrosion resistance

## Abstract

The high-energy ball milling process was applied to fabricate a composite material from 7075 aluminium alloy milling chips, silicon carbide, and titanium dioxide powders. Raw materials were ground, and the obtained powders were cold pressed and sintered. It was demonstrated that this method can be used in the recycling of aluminium alloy scrap characterised by a high surface-to-volume ratio, and also that chemical removal of the oxide layer from chips is not necessary. The finest particles, with 50 vol.% of their population below 36 μm, were obtained after grinding for 60 min at a 1000 rpm rotational speed. Such an intensive grinding was necessary to fabricate the compact composite material with a homogeneous microstructure and a low porosity of 0.7%. The corrosion resistance of the composites was studied in 3.5 wt.% NaCl solution using cyclic voltammetry and electrochemical impedance spectroscopy, and corrosion rates in the range of ca. 342 and 3 μA∙cm^−2^ were obtained. The corrosion mechanism includes aluminium alloy dissolution at the matrix/reinforcement interphase and around intermetallic particles localised within the matrix grains.

## 1. Introduction

Metal matrix composites, composed of relatively ductile metallic matrix and high-strength, often ceramic, reinforcement, are fabricated because of their better properties compared to the components alone. In this group, aluminium alloy matrix composites (AAMCs) are the most commonly applied. They offer better mechanical properties, such as strength, stiffness, and wear resistance, compared to aluminium alloys. Furthermore, their specific strength and specific stiffness are higher than those of many steels and titanium alloys, making them ideal for use in the aviation, maritime, and transport industries [1].

The current tendency to reduce CO_2_ emission in transportation involves, among other approaches, a widespread application of lightweight materials, such as aluminium alloys, but also aluminium alloy matrix composites. Unfortunately, aluminium, due to its high affinity to oxygen, is rather difficult to obtain from ore. When global consumption of the most important metals and the specificity of various metallurgical processes were taken into account, the environmental implications of their production from primary sources were estimated. Aluminium, in 2008, had the second highest global warming potential and the fourth highest negative influence on human health and the environment among the most commonly applied metals [2]. This high global warming potential is related to the emission of 1.1 Gt of CO_2_ equivalent per year, which constitutes 3% of the annual global emission of greenhouse gases. On the one hand, this emission could be significantly reduced because 67% of the electrical energy used for aluminium production is still obtained from fossil fuels [3]. On the other hand, the growing demand for aluminium will outweigh this positive trend [4]. Furthermore, the negative environmental impact of aluminium extraction and smelting, related to the emission of toxic substances, cannot be reduced in this way. The most hazardous by-products obtained at various stages of aluminium production include red mud, which is the highly alkaline residue from the Bayer process; the carbon lining of electrolytic cells, containing, among other components, fluorides and cyanides; salt slag from electrolysis; and gaseous products such as perfluorocarbons, hydrogen fluoride, polycyclic aromatic hydrocarbons, sulphur dioxide, carbon dioxide, methane, nitrous oxides, hydrofluorocarbons, and sulphur hexafluoride [5].

A promising strategy to reduce the negative environmental impact of aluminium production is to increase the amount of metal obtained from recycling. Aluminium scrap usually can be simply re-melted, and the energy expenditure related to this process is ca. 16 times smaller compared to the manufacture of aluminium from ore. Furthermore, the aforementioned hazardous substances are not produced. Therefore, the amount of recycled aluminium is expected to increase from the present one-third of its total production to about 50% in 2050. The three most important sources of aluminium post-consumer scrap are the building and construction industry, the packaging industry, and transport [3]. There are, however, certain forms of aluminium scrap that can be more difficult to recycle, for instance, machining chips.

Aluminium alloy chips are obtained during metal machining and constitute 13.7% of the waste in global manufacturing processes of this metal [6]. Their conventional recycling, i.e., melting, requires their prior consolidation. An alternative recycling strategy is a direct conversion of the chips into final products, where mainly extrusion and forging methods with subsequent sintering are applied. Powder metallurgy is a so-called semidirect method of processing of aluminium alloy chips and includes powder production, its subsequent consolidation, and sintering [6]. The latter approach can be applied not only for the fabrication of aluminium alloys, but is a convenient way to obtain aluminium alloy matrix composites.

On the one hand, powder metallurgy is regarded as expensive, because it requires raw materials in a powdery form [1]. On the other hand, the use of machining chips in this process makes it a cost-effective and environmentally friendly way of producing the final products. In addition, powder metallurgy processes have the advantage of avoiding problems with the wettability of the reinforcement with the liquid matrix, resulting in good adhesion at the matrix/reinforcement interphase [7].

Currently, the fabrication of aluminium alloy matrix composites from chips via the powder metallurgy route involves their mechanical grinding with reinforcement, cold or hot pressing, and sintering into a final product [8,9]. In this process, the main problem is obtaining a compact material due to insufficient fragmentation of the chips [10]. The presence of the aluminium oxide layer on the surface of the chips can also be an important issue during fabrication of AAMCs [11].

This problem can be overcome during high-energy milling. In general, high-energy milling, compared to conventional mechanical milling, has fewer limitations in the selection and volume fraction of the ground components, is still technologically uncomplicated, and offers a wide range of size of fabricated powders, from micro- to nanometres. Ground particles undergo plastic deformation; this causes accumulation of structural defects, strain hardening, and their subsequent fracturing. This helps to obtain a fine powder, favourable for shaping and sintering [12,13].

The degree of plastic deformation and fragmentation of the ground material depends on the milling time, the type of grinding balls, the rotation speed, and the type of reinforcement used. Typically, high-energy ball milling enables the production of composites with uniformly distributed reinforcement and good binding to the aluminium matrix, free of defects such as porosity, voids, and microsegregation [14,15,16,17,18]. It is important to note that despite the initial high fragmentation of the components, agglomeration of the particles may occur [12,19]. This may cause nonhomogeneity of the phase and chemical composition of obtained composites. The agglomeration of the component particles can be avoided via careful selection of milling parameters [20].

Many studies focused on the microstructure and properties of AAMCs obtained from powders made of aluminium [16,21], 2017 [15], 2024 [22], Al-Si [23], Al-Ni [24], 6082 [25], and 7075 aluminium alloys [26,27,28] in the conventional and high-energy milling processes. These processes were also applied to recycle machining chips or scrap from aluminium [19], Al-Si [29], Al-Cu-Mg [30], Al-Si-Cu [31], 6061 [32,33], and 7075 aluminium alloys [20,32,34]. Usually, the products of such a recycling process were composite materials. It was observed that their microstructure and properties were greatly affected by the size and shape of the chips. The important factor is also the surface cleaning method applied to the chips prior to grinding, and the properties of the reinforcement [8,30]. The typical reinforcing particles applied in AAMCs are Al_2_O_3_, SiO_2_, ZrO_2_, TiO_2_, ZnO, SiC, B_4_C, TiC, graphite, carbon nanotubes, Si_3_N_4_, BN, TiN, ZrB_2_, and TiB_2_ [1]. These particles not only determine the mechanical properties of the composite, but impact the grinding process itself by facilitating proper fragmentation of ground material [16]. In the case of machining chips, only a limited range of reinforcements was applied, such as multi-walled carbon nanotubes [20], SiC [31], Nb_2_Al, and glass bubbles [34]. It can be concluded that only a small fraction of studies on the application of mechanical milling in AAMC fabrication focuses on the machining chips.

The negative environmental impact of the metallurgical process can be limited to some extent by increasing the product lifetime. This strategy has the potential to reduce metal demand in developed countries, but its influence on demand in developing countries, with a high population growth rate, is limited [35]. However, its importance should increase with the increasing wealth of the inhabitants of currently developing countries. One of the most important factors that limits the longevity of metals is their corrosion. Therefore, numerous strategies of corrosion protection are applied in industry. Understanding the corrosion behaviour of newly developed materials is the first step toward their efficient protection.

AAMCs exhibit superior mechanical properties compared to aluminium alloys. This makes them especially attractive for transportation. The automotive industry mostly uses AMMCs for fabrication of pistons, engine blocks, and brakes, where wear resistance is more important than corrosion resistance. However, their application in structural elements of aircrafts and marine ship body parts may require a sufficient resistance to corrosion in chloride solutions [36]. Other possible applications, where traditionally aluminium alloys are applied and where their corrosion and wear resistance is ensured through protective coatings, are, e.g., components of hydraulic systems such as hydraulic gears and valves or components of aircraft undercarriage legs [37].

The corrosion behaviour of AAMC is related to the corrosion behaviour of its matrix. The latter, in turn, strongly depends on its chemical composition. In the case of the 7075 alloy, copper, magnesium and zinc are its main alloying elements. They dissolve to some extent in the matrix and, together with impurities such as iron and silicon, constitute intermetallic particles. When dissolved in the matrix, Cu shifts the corrosion potential in the positive direction, while Mg and Zn shifts it in the negative direction, compared to unalloyed aluminium [38]. Intermetallic particles containing iron and copper, such as Al_7_Cu_2_Fe, and (Al,Cu)_6_(Fe,Cu) are more noble than the matrix and start its corrosion. Mg_2_Si and MgZn_2_ undergo dissolution because they are less noble compared to the matrix. The dissolution of the latter may cause intergranular corrosion [38]. Nonmetallic particles, for example SiC, TiO_2_, and Al_2_O_3_, commonly used as reinforcement in AAMCs, can affect their corrosion behaviour, but this influence is not always straightforward. Nonmetallic particles are often regarded as electrochemically inert components of metal matrix composites that decrease their effective corrosion area and the corrosion rate [39]. This was observed for the 7075 alloy reinforced with SiC [39], SiC and graphite [40], SiC and Al_2_O_3_ nanoparticles [41], garnet [42], red mud [43], short basalt fibres [44], and Al_2_O_3_ [45]. It should be noted that in the case of SiC, also no significant improvement in the corrosion resistance of the composite was reported [46]. The influence of TiC on the corrosion behaviour of 7075-based AAMC was unclear [47]. TiO_2_ generally decreased the rate of anodic reaction but increased the rate of the cathodic [48]. Addition of B_4_C [46] and Al_2_O_3_, in turn, was detrimental to the corrosion resistance of the composite material [49]. When CeO_2_ and MoS_2_ were added together to the 7075 alloy matrix, a lower corrosion rate was obtained when ceria was the dominating reinforcement [50].

Most of the reported corrosion studies were conducted in 3.5 wt.% NaCl solution, where the 7075 alloy may undergo pitting corrosion. It was observed that SiC and Al_2_O_3_ do not change the pitting potential of AAMC, compared to the matrix. However, pitting corrosion of a composite material, if it occurs, may be more detrimental than that of the alloy, due to the formation of crevices at the matrix/reinforcement interphases [51]. These crevices further accelerate the corrosion process, due to non-uniform access of oxygen to the surface. Thus, enhanced contact in the matrix/reinforcement interphase should be beneficial to the corrosion resistance of the composite material [46]. This makes high-energy ball milling a promising method for the fabrication of aluminium alloy matrix composites from chips.

The aim of this work was to obtain composite materials from 7075 aluminium alloy machining chips, silicon carbide, and titanium dioxide in the high-energy ball milling process and to study the influence of grinding parameters on their microstructure and corrosion behaviour. The 7075 alloy was selected due to its wide industrial application. Chips obtained during the machining of this alloy can be an important source of raw material for composite fabrication. Furthermore, this alloy offers the possibility of increasing the strength of the composite material through its heat treatment. SiC was chosen because of its high hardness. TiO_2_ can be relatively easily obtained in the form of fine powder. It was expected that the combination of reinforcement particles of different sizes would help to obtain a nonporous composite material. The grinding parameters tested in this work included the grinding time, rotation speed, and the content of the ceramic particles. The characteristic features of the applied high-energy ball milling process were a relatively short milling time, 1 h, cold pressing of the powders obtained prior to sintering instead of the commonly used hot process, and no need for chemical pretreatment of the aluminium alloy chips to remove the oxide layer. The results obtained constitute a basis for future fabrication of the composite material from 7075 aluminium alloy machining chips and characterisation of their mechanical properties and wear resistance.

## 2. Materials and Methods

**Figure 1:**
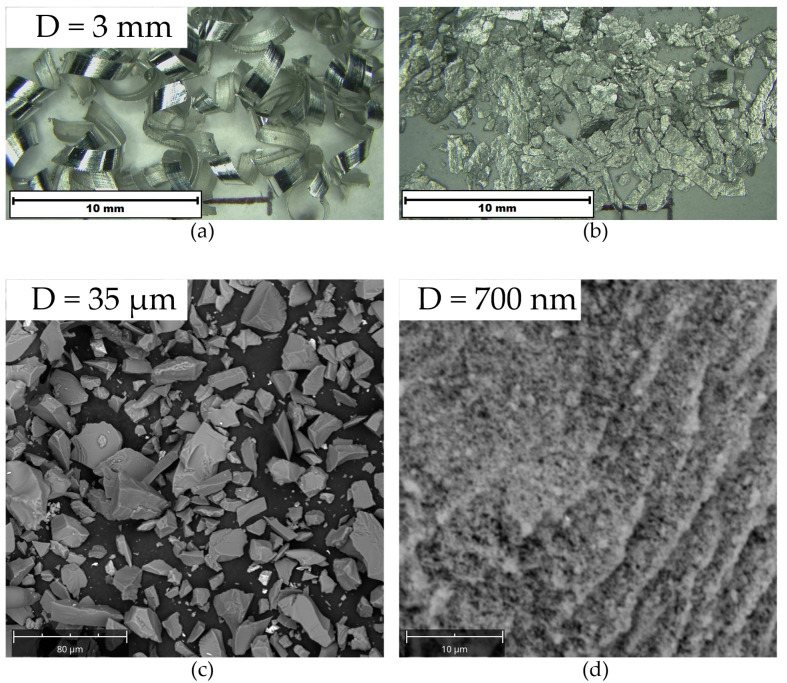
Morphology of components used for the fabrication of the composite material: (**a**) 7075 aluminium alloy chips; (**b**) 7075 aluminium alloy chips after pre-grinding; (**c**) SiC powder; (**d**) Ti_2_O powder.

The composite material was fabricated from 7075 T6 aluminium alloy chips, which formed its matrix, and SiC and TiO_2_ ceramic particles as reinforcement. Chips were obtained during the milling of the aluminium alloy sheet, with dimensions of 8 × 100 × 100 mm (Figure 1a). The 7075 aluminium alloy sheet was purchased from Adamet-Niemet Rzeszow, Poland. Its nominal chemical composition is shown in Table 1. Before composite fabrication, the chips were cleaned in acetone using an ultrasonic cleaner (Sonoswiss AG, Ramsen, Switzerland) for 30 min and then dried for 6 h at a temperature of 80 °C. The oxide layer formed during mechanical machining was not removed. The chips were then pre-ground in a Siemens Herzog mill (Herzog Maschinenfabrik GmbH & Co. KG, Osnabrück, Germany for 1 min to facilitate subsequent composite formation (Figure 1b). Reinforcing particles of various shapes and sizes (Figure 1c,d), obtained from Chempur, Piekary Śląskie, Poland, were used to obtain the composite material. All other chemical reagents used were also purchased from Chempur.

The composite materials were produced in the high-energy ball milling process using the Pulverisette 7 Premium Line Fritsch planetary mill (Fritsch GmbH Mahlen und Messen, Idar-Oberstein, Germany). Aluminium alloy chips were ground together with ceramic particles of SiC and TiO_2_. The total quantity of ceramic particles was 3, 6, and 12 wt.%, and the weight ratio of silicon carbide and titanium dioxide was 1:1 (Table 2). The total mass of the milled materials was always 13 g. The grinding process was carried out in an 80 mL grinding bowl lined with Al_2_O_3_ using 5 mm diameter Al_2_O_3_ grinding balls. The ratio of the mass of the grinding balls to the mass of the components of the composite powder was fixed during all experiments and equal to 4:1. The grinding took place in an inert argon atmosphere with the addition of 2 wt.% of stearic acid. Before grinding, the bowl with the chips and the ceramic particles was purged with argon for 15 min. The powders were ground for 5 min and then cooled for 30 min in a closed bowl. A grinding and subsequent free cooling step constituted one grinding cycle. The composite materials were obtained in 6 or 12 grinding cycles. When the process was finished, the obtained powders were sealed in vacuum bags. The milling parameters are summarised in Table 2.

The lower value of the grinding speed corresponds to those commonly used in the literature, where values between 200 and 600 rpm are reported, for instance, 200 rpm in [31], 240 rpm in [21], 250 in [20,26], 350 rpm in [24,53], 300 rpm in [22,30], 450 in [27], and between 400 and 600 in [15]. A high grinding speed was applied more rarely, e.g., in [16,23], and never in the case of machining chips. It was expected that a relatively high grinding speed would be necessary to achieve a very fine composite powder, essential for the production of a compact composite material. Consequently, when relatively low grinding speed were applied, chip processing was rather prolonged, e.g., 20 h in [53], between 18 and 96 h in [19], and 40 h in [31], but also values comparable to those applied in this work were reported [20,34].

The particle size distribution of powders obtained at a rotational speed of 400 rpm was obtained from sieve analysis, using a Fritsch Analysette 3 Spartan vibrating shaker (Fritsch GmbH Mahlen und Messen, Idar-Oberstein, Germany). The powders were sequentially sifted through sieves with mesh sizes of 900, 800, 560, 400, 300, and 200 μm. The smallest particles, with diameter < 200 μm, were further analysed using the laser diffraction method with an IPS U particle size analyser (Kamika Instruments Warsaw, Poland). The same method was applied for powders obtained at a grinding speed of 1000 rpm. The particle size was analysed in the range between 3.2 and 523 μm. The particles were classified into 64 size classes, with the size range within each class ca. 8 μm. Those outside the aforementioned range were included in the external classes. The average value of the particle size, i.e., the middle of each class, is presented in graphs. The volume fraction of each class was calculated as the average of 3 measurements. The uncertainties of the volume fractions were calculated using Equation (1):

(1) $$\Delta V=\frac{t_{0.95,\;n-1}\cdot s_{V}}{\sqrt{n}}$$

where *S*_V_ is the standard deviation, *n* = 3 is the number of measurements, and *t*_0.95, *n*−1_ is the Student’s t factor for the 95% confidence level.

The morphology of the fabricated powders was observed using a Phenom XL scanning electron microscope (SEM) with a backscattered electron detector (BSE) (Thermo Fisher Scientific Inc., Waltham, MA, USA).

The powders obtained were then pressed into pellets of ca. 5 mm height and 15 mm diameter, using a Siemens Herzog press (Herzog Maschinenfabrik GmbH & Co. KG, Osnabrück, Germany) at a force of 100 kN. Each pellet was pressed for 5 min. Subsequently, they were sintered in an argon atmosphere using a Carbolite tube furnace (Carbolite Gero Ltd., Hope Valley, UK), and the composite materials in the bulk form were obtained. The pellets were first heated in the furnace to a temperature of 550 °C and annealed at this temperature for 1 h. The temperature was then raised to 580 °C, and the pellets were heated for the next 3 h. Finally, they were cooled in the furnace in an argon atmosphere. The selection of the sintering temperature was based on the literature, where the temperature range between 550 °C and 600 °C was found as suitable [54,55]. This was further verified experimentally. Initial tests carried out for the powder obtained at a grinding speed of 400 rpm indicated that proper sintering was impossible at a temperature below 550 °C. On the contrary, the use of higher temperatures, such as 600 °C for powders ground at a speed of 1000 rpm, led to local melting of the sintered material surface. A two-stage sintering process prevented the formation of cracks that were observed when one-stage sintering was applied.

The microstructure of the composite materials obtained was assessed by metallographic sectioning. The specimens were cut in half using a Struers Labotom-5 metallographic cutter, embedded in conductive resin using a Struers Citopres-5 press (both from Struers, Ballerup, Denmark) and then gradually ground and polished using an ATM Saphir 330 double disc grinder and polisher (ATM Qness GmbH, Mammelzen, Germany). The grinding was carried out on increasing grit sandpaper: 320, 500, and 1200. After grinding, the samples were polished using polycrystalline diamond suspensions with a grain size of 3 and 1 μm. Microstructure observations were made on unetched specimens.

The specimens were examined using a Phenom XL scanning electron microscope with a BSE detector. Chemical composition analysis in the micro-areas of the specimens was conducted using an X-ray energy dispersion spectrometer integrated with a Phenom XL SEM microscope, operating at an accelerating voltage of 15 kV. The results of these analyses are presented as maps showing the relative content of elements in the studied areas.

The porosity of the selected composites was quantitatively analysed using a computer-aided method. Their SEM images, ten for each analysed specimen, were converted into binary measurement-oriented images. This process involved enhancing the contrast, performing histogram equalisation, and applying median smoothing to the SEM images. Subsequently, manual binarisation was conducted, and the resulting binary images of porosity were further refined manually. The pores were then automatically detected and quantified using Leica Application Suite v3.7 image analysis software.

The corrosion behaviour of the composite materials was studied in 3.5 wt.% NaCl aqueous solution, in equilibrium with air, at *T* = 30 °C. Solutions were stagnant during measurements; each corrosion test was performed in 100 mL of the solution. A standard three-electrode electrochemical cell connected to an SP-300 potentiostat (Bio-Logic SAS, Seyssinet-Pariset, France) was applied; all experiments were conducted in the Faraday cage. Working electrodes were prepared from composite materials. The front sides of each specimen were ground using emery papers, of 320, 500 and 1200 grit, and polished with diamond suspensions, of 3 and 1 μm. A thin aluminium rod was attached to the backside of the specimen using a silver adhesive paste. The entire electrode, except for the polished surface, was covered with protective coating (Henkel Poland, Sp. z o.o., Warsaw, Poland). The surface area of the working electrodes was 1.33 cm^2^. Silver wire electrochemically coated with silver chloride, immersed directly in the chloride solution, served as the reference electrode. All potential values in this work are provided vs. the Ag|AgCl (KCl sat.) electrode, knowing that the potential of Ag|AgCl wire in 3.5 wt.% NaCl at 30 °C was +48 mV vs. the Ag|AgCl (KCl sat.) electrode. A spring made of platinum wire was applied as the counter electrode.

The open circuit potential (OCP) of the composite materials studied was measured for 22.3 h. Then, their electrochemical impedance spectra were measured in the frequency domain between 200 kHz and 1 mHz, with the root mean square of the applied sinusoidal perturbation of 5 mV. Subsequently, two types of cyclic voltammograms were recorded. In the case of the first, the starting potential was −15 mV vs. OCP, the reverse potential +15 mV vs. OCP, the finishing potential at the starting potential, and the potential scanning rate 5 mV∙min^−1^. It was used to estimate the corrosion rate. The second one was used to study the behaviour of the composite materials during anodic polarisation. It was recorded from the OCP, with the scanning rate of 10 mV∙s^−1^. The scan was reversed when the anodic current density of 5 mA∙cm^−2^ was reached, and finished back at the OCP. The potential drop in the electrolyte between the working and reference electrodes, as a function of current density, was calculated for both cyclic voltammograms using the value of electrolyte resistance determined from the impedance spectra at 200 kHz. All cyclic voltammograms present the potential values corrected for this potential drop.

**Figure 2:**
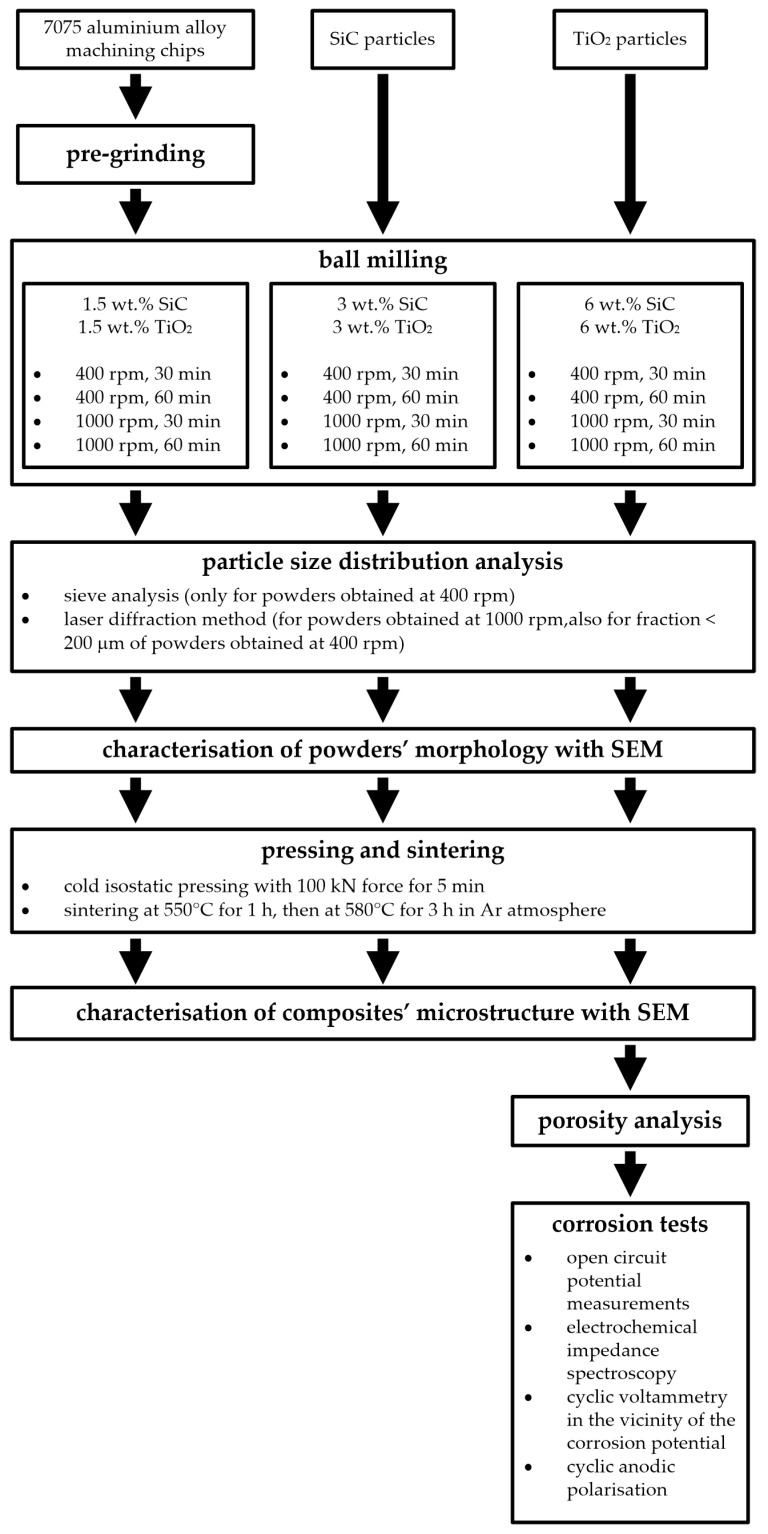
Fabrication and characterisation of the composite materials.

The most important aspects of the fabrication and characterisation of the composite materials are summarised in Figure 2.

## 3. Results and Discussion

### 3.1. The Influence of Grinding Parameters on the Morphology of Obtained Powders

**Figure 3:**
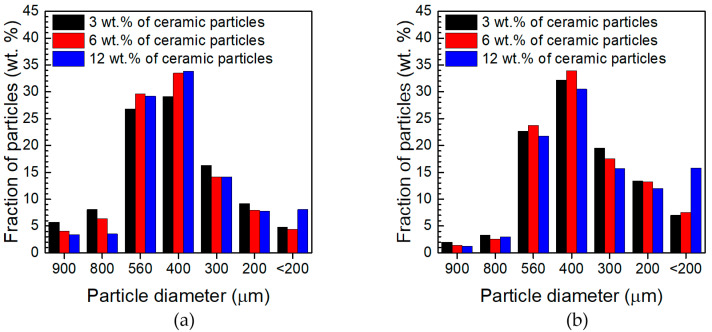
Particle size distribution obtained from the sieve analysis of powders fabricated at 400 rpm grinding speed: (**a**) total grinding time 30 min, (**b**) total grinding time 60 min.

Visual inspection of the obtained powders indicated that the adopted grinding parameters strongly influenced the particle size. In particular, the low grinding speed resulted in obtaining relatively coarse particles. Thus, the first step of characterising the morphology of the obtained powders was sieve analysis. It was conducted for powders obtained at a grinding speed of 400 rpm. When the grinding time was 30 min, the vast majority of the particles had a diameter in the range between 800 and 400 μm (Figure 3a). When the grinding time was extended, the number of particles greater than 560 μm decreased, while the number of particles with diameter less than 400 μm increased (Figure 3b). Although SiC particles, which are significantly harder compared to the aluminium alloy, should facilitate the fragmentation of the chips, no clear correlation was found between the quantity of ceramic powders and the average particle diameter. Only in the case of the smallest particles their weight fraction in the powder obtained increased as the total amount of ceramic particles increased.

**Figure 4:**
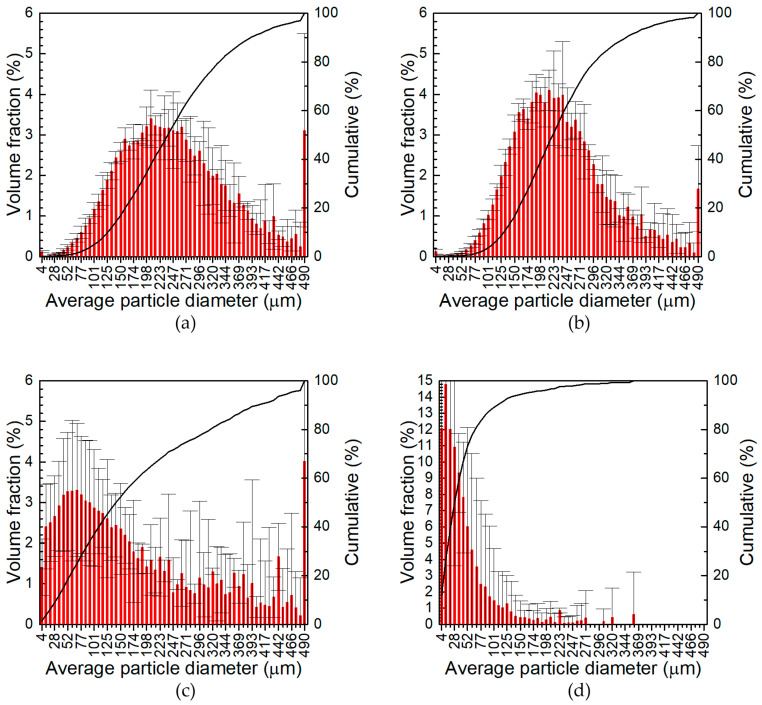
Particle size distribution of powders obtained from high-energy ball milling of 7075 aluminium alloy chips with 1.5 wt.% SiC and 1.5 wt.% TiO_2_: (**a**) for 30 min at 400 rpm; (**b**) for 60 min at 400 rpm; (**c**) for 30 min at 1000 rpm; (**d**) for 60 min at 1000 rpm.

**Figure 5:**
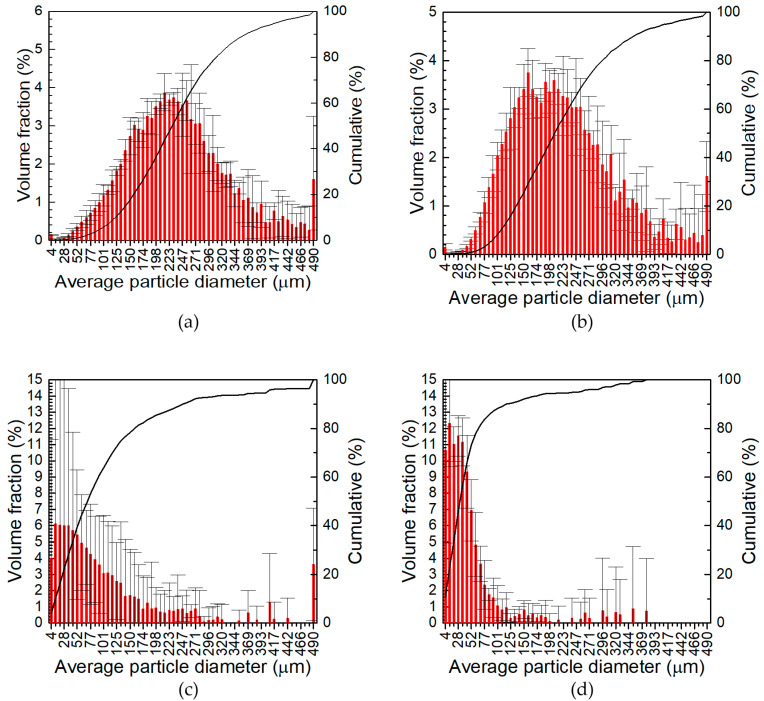
Particle size distribution of powders obtained from high-energy ball milling of 7075 aluminium alloy chips with 3 wt.% SiC and 3 wt.% TiO_2_: (**a**) for 30 min at 400 rpm; (**b**) for 60 min at 400 rpm; (**c**) for 30 min at 1000 rpm; (**d**) for 60 min at 1000 rpm.

**Figure 6:**
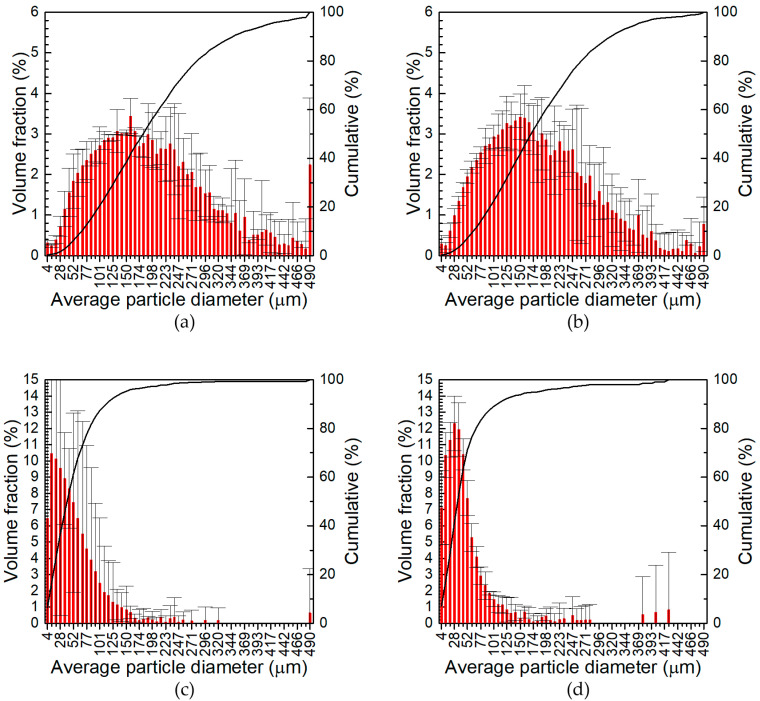
Particle size distribution of powders obtained from high-energy ball milling of 7075 aluminium alloy chips with 6 wt.% SiC and 6 wt.% TiO_2_: (**a**) for 30 min at 400 rpm; (**b**) for 60 min at 400 rpm; (**c**) for 30 min at 1000 rpm; (**d**) for 60 min at 1000 rpm.

The size distribution of the powders obtained at 1000 rpm grinding speed, as well as the finest fraction of powders fabricated at 400 rpm, that is, particles with diameter <200 μm, collected at the receiver of the sieve shaker, was determined using the laser diffraction method (Figure 4, Figure 5 and Figure 6). First, it can be observed that in the case of powders obtained at 400 rpm, there is still a significant volume fraction of particles >200 μm, between 40 and 65 vol. %, which was attributed to their elongated shape with one dimension below 200 μm and the other >200 μm. The elongated shape of these particles was further confirmed in microscopic analysis. The volume fractions of particles with average diameter <4 μm and >490 μm were added to the volume fractions of particles with average diameter equal to 4 μm and 490 μm, respectively. The contribution of these large particles, i.e., >490 μm, was significant in the case of all powders obtained at 400 rpm (Figure 4a,b, Figure 5a,b and Figure 6a,b), as well as powders ground at 1000 rpm for 30 min, when the total amount of ceramic particles introduced to the grinding process was 3 and 6 wt.% (Figure 4c and Figure 5c). The contribution of the smallest particles, i.e., with average diameter below 4 μm, was important in the case of all the powders obtained at 1000 rpm grinding speed.

The second interesting observation is related to the magnitude of the error bars in Figure 4, Figure 5 and Figure 6. It is relatively low for the powders manufactured at a grinding speed of 400 rpm, regardless of the grinding time and the quantity of ceramic particles in the grinding bowl, (Figure 4a,b, Figure 5a,b and Figure 6a,b), as well as for the two powders obtained at a grinding speed of 1000 rpm and a grinding time of 60 min with a total content of ceramic particles of 6 and 12 wt.% (Figure 5d and Figure 6d). In other cases, the magnitude of the error bars was high. The high uncertainty of the volume fraction of the particles is probably related to the relatively wide distribution of the particle size and may be attributed to incomplete grinding. When the grinding time increased, the particle size distribution narrowed and the magnitude of error bars decreased (Figure 5c,d and Figure 6c,d).

**Figure 7:**
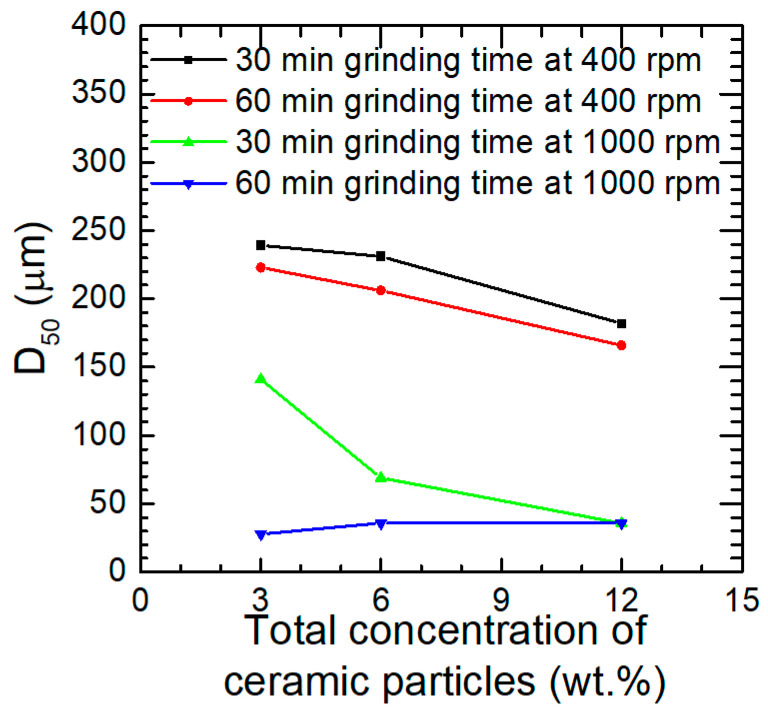
D_50_ diameter of the powders obtained under different grinding conditions.

Third, it can be concluded that the particle size distribution became narrower and the average particle size decreased as the total content of the ceramic particles, the grinding time, and the grinding speed increased. It was found that the grinding speed had the most significant impact on the particle size of the powders obtained. The same was also observed by Witkowska et al. [56]. The role of the grinding parameters can be better visualised using D_50_ average particle diameter, i.e., the diameter of the particles obtained from the particle size distribution at 50% of the cumulative volume (Figure 7). When the grinding speed was 400 rpm, D_50_ only slightly decreased with prolongation of the grinding time, while a greater impact was observed with increasing the total concentration of ceramic particles. The strongest influence of the grinding time on D_50_ was observed at a grinding speed of 1000 rpm, when the total content of ceramic particles in the grinding bowl was 3 wt.%. Rojas-Díaz et al. showed three stages of the grinding process of aluminium chips. In the first stage, the size of the ground particles decreased, and then, in the second stage, it stabilised. This means that increasing the grinding time does not significantly affect the particle size. In the third stage, further fragmentation of particles occurred. The average particle size in the second grinding stage at the 55 rpm grinding speed was around 50 μm [19]. A similar effect was observed in the case of 7075 aluminium alloy powder and carbon nanotubes as reinforcement. In this case, after 1 h of grinding at 450 rpm, an average diameter of 30 μm was obtained [27]. It was also observed that a too-long grinding time leads to agglomeration of powder due to cold welding of the particles [19,20]. The role of ceramic particles in the grinding process was again the most obvious for the powders obtained at 1000 rpm and 30 min. Because these particles are harder than the aluminium alloy, they facilitate the fragmentation of the chips. The influence of the ceramic particles on the particle size was insignificant when the grinding time was sufficiently long, in our case 60 min.

On the one hand, the primary role of the reinforcement is to increase the strength, hardness, and the wear resistance of the composite material. These properties are controlled through the addition of the appropriate quantity of reinforcing particles, although the strain hardening of the chips during grinding can also play a role. On the other hand, fine grinding of the aluminium alloy chips is necessary to obtain compact composite material, as is shown in the following section of the article. Therefore, preferable grinding conditions are such when a relatively fine powder can be obtained regardless of the concentration of the ceramic particles. In our case, only the grinding speed equal to 1000 rpm together with the grinding time of 60 min fulfilled this condition.

**Figure 8:**
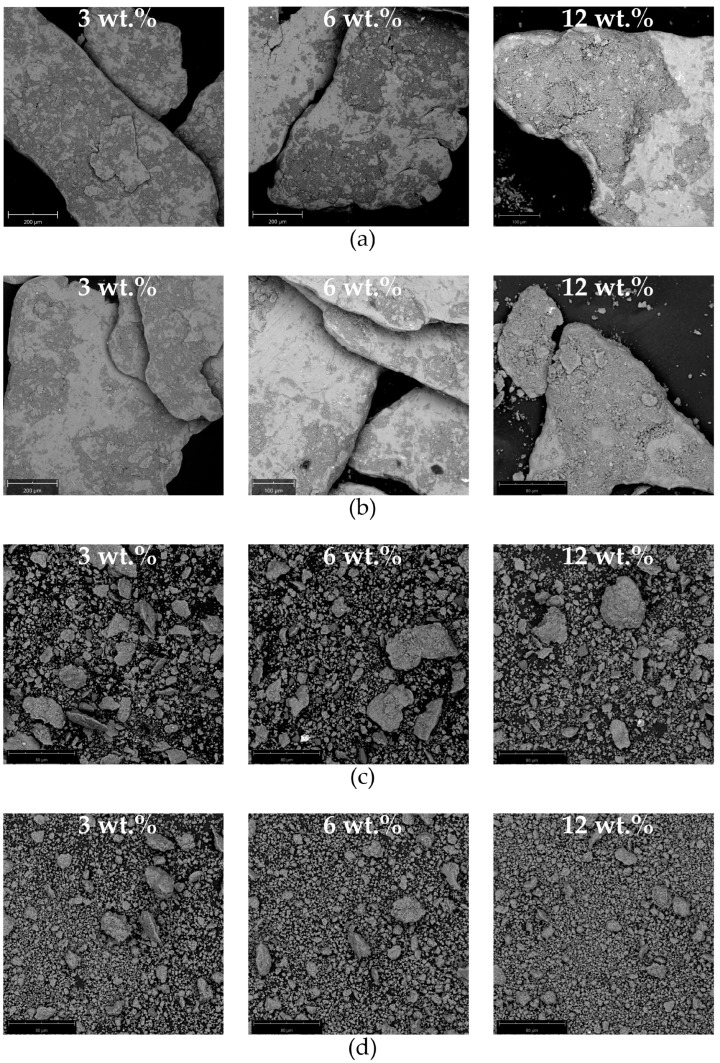
Morphology of the powders obtained as a function of the total concentration of ceramic particles: (**a**) grinding for 30 min at 400 rpm; (**b**) grinding for 60 min at 400 rpm; (**c**) grinding for 30 min at 1000 rpm; (**d**) grinding for 60 min at 1000 rpm.

SEM analysis confirmed the influence of the grinding speed on the particle size of the powders obtained. When it was equal to 400 rpm, relatively large aluminium alloy particles of elongated, flake-like shape were obtained. Their morphology neither changed significantly when increasing the total concentration of ceramic particles, nor upon increasing the grinding time from 30 to 60 min (Figure 8a,b). Dias et al. observed, in the case of aluminium bronze, that at a grinding speed of 300 rpm, the grinding time necessary for transformation of the chips into the equiaxial particles was 30 h [57]. It can also be observed that the aluminium alloy particles are enveloped by a top layer consisting of a mixture of small reinforcement particles of various shapes (Figure 8a,b). This effect is due to the significant difference in size between the aluminium alloy chips and the reinforcement particles. The same was observed by Gasha et al. after 2 h of grinding of 2017 aluminium alloy powder with SiC particles [15], and by Suśniak et al. [31].

The morphology of the powders changed significantly when the grinding speed increased to 1000 rpm (Figure 8c,d). Extensive fragmentation of the aluminium alloy chips occurred, regardless of the grinding time, and no large or elongated particles were found. Rounding of the sharp edges of the particles was also observed. The influence of the grinding time is more clearly visible in the case of high than low grinding speed. This stays in agreement with the data presented in Figure 7, which shows a significant difference between the average diameter of D_50_ of powders ground for 30 and 60 min at 1000 rpm and the low and medium total content of the ceramic particles.

### 3.2. Microstructure of Composite Materials

**Figure 9:**
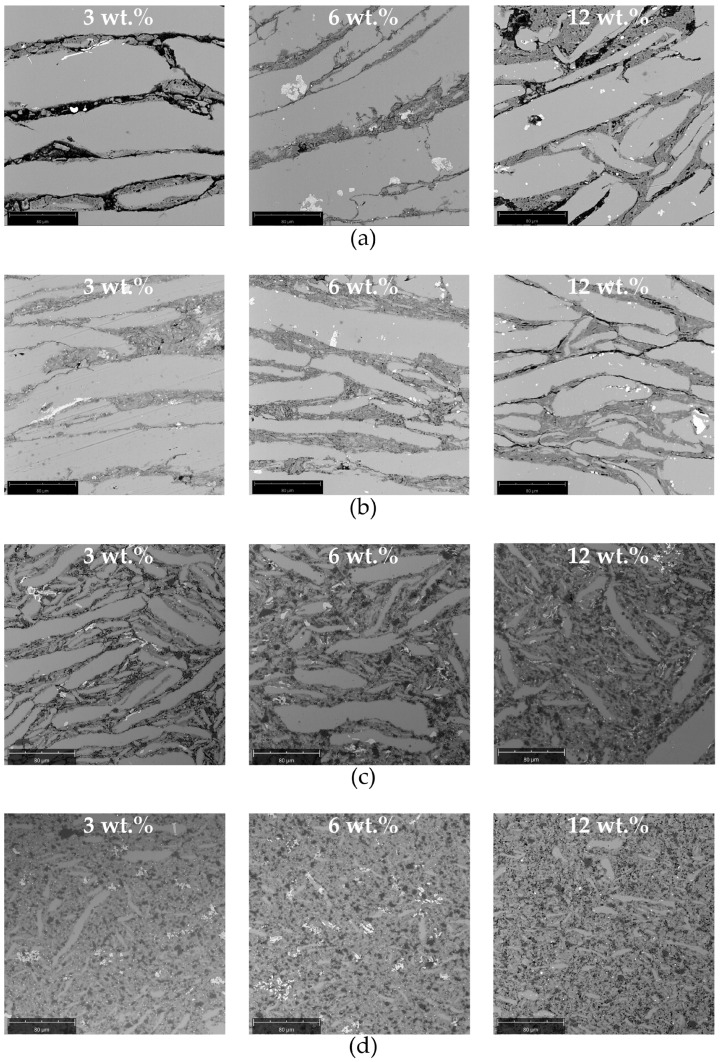
Microstructure of composite materials obtained after cold isostatic pressing and sintering: as a function of the total concentration of ceramic particles: (**a**) grinding for 30 min at 400 rpm; (**b**) grinding for 60 min at 400 rpm; (**c**) grinding for 30 min at 1000 rpm; (**d**) grinding for 60 min at 1000 rpm.

The 7075 aluminium alloy matrix composite materials in bulk form were obtained after cold isostatic pressing and sintering. Microscopic characterisation revealed a laminar microstructure of the materials obtained during grinding at 400 rpm (Figure 9a,b). The matrix grains are elongated and arranged parallel to each other with ceramic particles between them. The latter occurred because the aluminium alloy chips were covered with ceramic particles (Figure 8a,b). Relatively thick ceramic layers that separate matrix grains are unfavourable in terms of the mechanical properties of composite materials. Due to different stress–strain behaviour of the matrix and reinforcement, nucleation of cracks and their propagation along the matrix/reinforce interphase would occur. Therefore, rather low fracture toughness is expected. The wear resistance of such a composite material would also be low. Cracking of the reinforcement under pressure would result in removal of ceramic particles. These particles, which are much harder than the matrix, would significantly increase the wear rate of the composite material and/or its counterpart. The parallel arrangement of the matrix grains was disturbed when the total concentration of ceramic particles increased. This was because the particles facilitated the fragmentation of the chips during grinding. When the grinding time was extended from 30 to 60 min, the aluminium alloy grains became narrower, but the microstructure was still unfavourable due to the large quantity of ceramic particles between the matrix grains. This improved only slightly when the quantity of ceramic particles increased (Figure 9b).

When the grinding was carried out at 1000 rpm for 30 min, the laminar microstructure was not obtained. There were elongated matrix grains, but they became randomly oriented and their size was much smaller compared to those from the lower grinding speed. However, certain porosity and nonuniform reinforcement distribution were visible between the matrix grains (Figure 9c). In this case, the role of reinforcement in the grinding process was clearly visible. When the quantity of ceramic particles increased, the size of the aluminium alloy grains decreased. It was observed that increasing the grinding time to 60 min led to further fragmentation of the grains of the aluminium alloy and overall homogenization of the material (Figure 9d). In this case, the influence of the quantity of ceramic particles on the microstructure of the composite material was hardly visible. This remains in agreement with the results of the analysis of the particle size distribution. The obtained microstructure seems to be the most favourable among those obtained in terms of the mechanical properties of the composite material. It decreases the risk of brittle fracture along the matrix grains, offering also uniform hardness and wear resistance compared to the materials obtained from powders manufactured at a 400 rpm grinding speed. Finally, it offers the possibility of tuning the mechanical properties of the composite materials via the addition of an appropriate quantity of ceramic particles to the grinding step, without affecting its microstructure. It should also be noted here that the strength of such a composite material could be increased, not only by increasing the content of the reinforcement but also by heat treatment. That is because 7075 aluminium alloy can be significantly strengthened due to precipitation hardening.

The results obtained indicate the possibility of fabricating the composite material from aluminium alloy chips via high-energy milling and cold pressing. This has already been reported for aluminium [19], AlSi5Cu2 aluminium alloy [31], 7075 aluminium alloy [34], and 6061 aluminium alloy [53]. However, in our case, it seems that a low porosity can be obtained with relatively short grinding time. It can be concluded that the high-energy input provided during the grinding at 1000 rpm reduces the time necessary for a sufficient fragmentation of the aluminium alloy chips. The reduction in the difference in the size and shape of the components enabled a dense packing of the powder particles during pressing. This, in turn, was necessary for the effective sintering and formation of the compact composite material.

**Figure 10:**
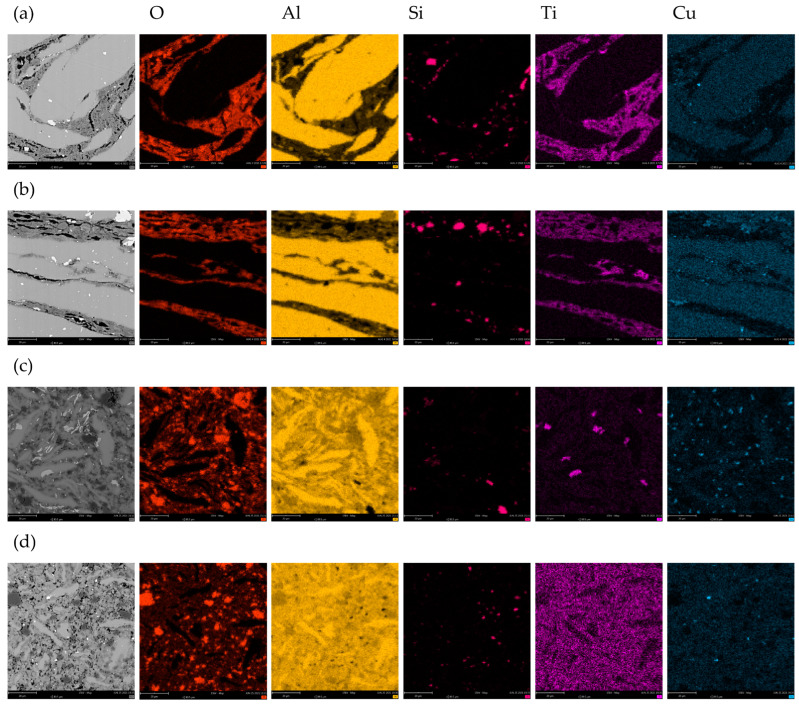
Chemical composition (EDS mapping) of composite materials obtained from powders ground: (**a**) for 30 min at 400 rpm; (**b**) for 60 min at 400 rpm; (**c**) for 30 min at 1000 rpm; (**d**) for 60 min at 1000 rpm; total concentration of ceramic particles was 12 wt.%.

The further characterisation of the composite materials was restricted to those fabricated from powders ground with 12 wt.% of the ceramic particles. These composites have the most promising microstructure in terms of homogeneity and porosity for a given set of fabrication parameters, i.e., grinding speed and time. Certain additional information on the mechanism of formation of composite materials could be obtained from the analysis of their chemical composition. First, in the case of composite materials obtained at the 400 rpm grinding speed, the grains of the aluminium alloy are separated with the layers of TiO_2_; within these layers, relatively large particles of SiC were found (Figure 10a,b). In addition, few copper-rich intermetallic particles were observed within the matrix. When the grinding speed was increased to 1000 rpm, the chemical composition of the composite materials became more homogeneous (Figure 10c,d), with much smaller areas enriched with oxygen and titanium; also, the silicon carbide particles were fragmented, especially when the grinding time was 60 min (Figure 10d). When the grinding time was 30 min, titanium-rich phases were observed between the grain boundaries of the matrix. This suggests a reaction between TiO_2_ particles and the matrix and the formation of a presumably TiAl intermetallic phase (Figure 10c). These phases were not visible when the grinding time was 60 min. This may indicate that they were ground and uniformly distributed within the microstructure. It is interesting that the areas with relative high oxygen concentration and low titanium content can be identified in Figure 10c,d. Therefore, they cannot be identified as TiO_2_. It can be speculated that oxygen is in the form of Al_2_O_3_. This might indicate that the aluminium oxide layer initially present on the chips was fragmented and agglomerated during grinding. Possible removal of Al_2_O_3_ from the chip surface during high-energy grinding should facilitate obtaining a compact material after sintering. In addition, the aluminium oxide particles, distributed between the aluminium alloy grains, may constitute additional reinforcement of the composite material. This has not been reported in the literature yet and deserves further studies. Cu-rich intermetallic particles were observed in the matrix of all of the discussed composite materials. This constitutes another interesting field of further studies. The high temperature during grinding and subsequent sintering probably ensures the equilibrium microstructure within the aluminium alloy grains, with sub-micrometre hardening particles coarsened. Thus, improvement of the hardness of these composite materials can be obtained not only with the addition of ceramic particles at the grinding stage, but also after precipitation hardening of the sintered material.

**Figure 11:**
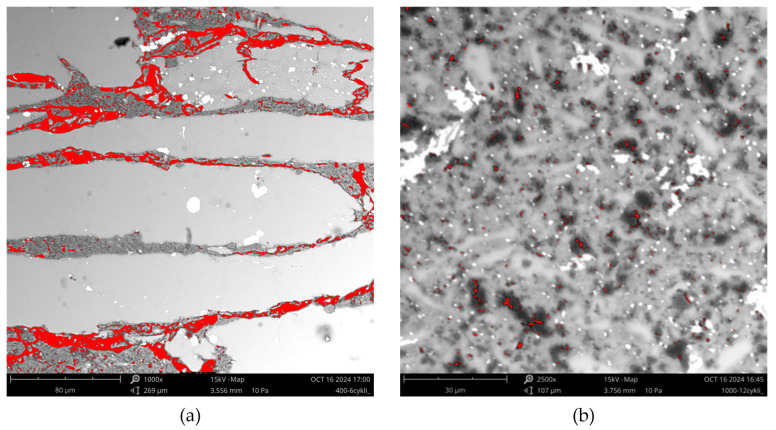
The examples of SEM images showing the detected porosity of the composite materials: (**a**) ground for 30 min at 400 rpm; (**b**) ground for 60 min at 1000 rpm.

The determination of porosity was the final step in characterising the microstructure of the composite materials obtained. A significant difference in the microstructure of the materials obtained at the different grinding speed (Figure 9) made the assessment of their porosity impossible on the images obtained at the same magnification. Therefore, the porosity analysis of the composite materials obtained at a grinding speed of 400 rpm was carried out at a magnification of 1000 times (Figure 11a). In the case of powders ground at a speed of 1000 rpm, SEM images made at a higher magnification, i.e., 2500 times, were used (Figure 11b). This allowed for the accurate detection of the porosity of the studied materials. Computer image analysis revealed that materials obtained at a speed of 1000 rpm for 60 min had the lowest porosity, of 0.7%. On the contrary, the highest porosity was observed in the composite made of powder ground at 400 rpm for 30 min (Table 3).

### 3.3. Corrosion Behaviour of the Composite Materials

#### 3.3.1. Corrosion Potential

**Figure 12:**
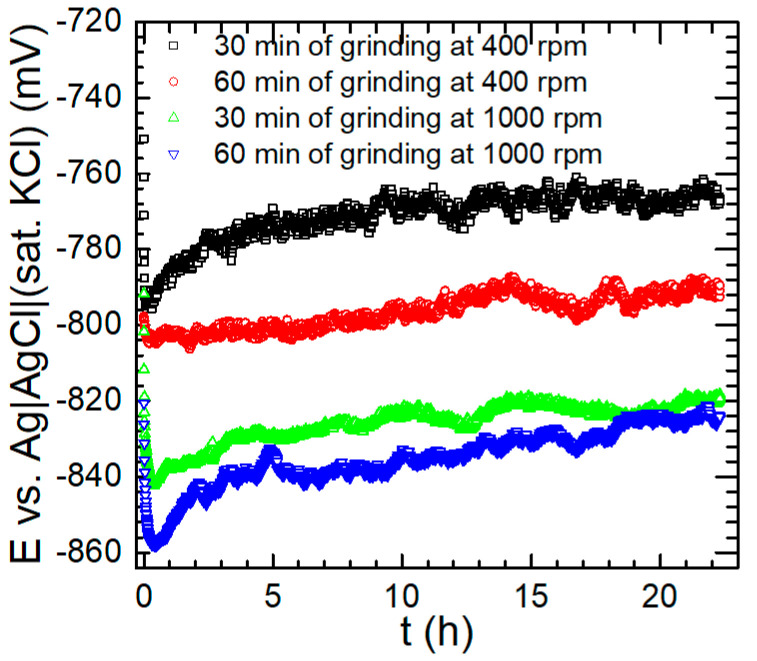
Open circuit potential of the composite materials immersed in 3.5 wt.% NaCl solution, in equilibrium with air, *T* = 30 °C, as a function of their fabrication conditions, i.e., grinding speed and total grinding time for the total concentration of ceramic particles 12 wt.%.

Open circuit potential (OCP) measurements are one of the simplest in corrosion studies. The evolution of OCP with time corresponds to changes in cathodic and anodic reaction rates in the corrosion cell, e.g., due to dissolution or formation of a protective layer. When the studied system achieves the stationary state, the OCP remains stable over time and is then called the corrosion potential. Achieving a stationary state is necessary for a more sophisticated analysis of the corrosion process. Corrosion potential values can also show the tendency of the studied material to corrosion in a given environment. However, this should be additionally verified during further electrochemical studies. In the case of aluminium alloys, their corrosion potential is highly dependent on their chemical composition and metallurgical condition [38]. The same is true for aluminium alloy matrix composites [48]. The evolution of the OCP of the composite materials with time is shown in Figure 12. The data recorded during the last 2 h of the experiment were averaged and the corrosion potential *E*_corr_ was obtained. It decreased as the composite fabrication process became more intensive. For 30 min of grinding at 400 rpm, −766 mV vs. the Ag|AgCl (sat. KCl) reference electrode was obtained. This value decreased to −791 mV when the grinding time was doubled. A further decrease was observed to −820 mV for the rotational speed of 1000 rpm and 30 min of grinding. The subsequent increase in the grinding time to 60 min had only a minor impact on *E*_corr_ and −825 mV was obtained. It can be noted that these results are comparable to those reported by Andreatta et al. for various tempers of the 7075 alloy in 3.5 wt.% NaCl at pH = 4 [38], i.e., between −716 mV and −810 mV vs. the Ag|AgCl (sat. KCl) reference electrode. Rodiĉ and Miloŝev in a 0.6 wt.% NaCl solution obtained a slightly higher value, that is, −656 mV vs. the Ag|AgCl (sat. KCl) reference electrode [58]. In the case of 7075 alloy matrix composite reinforced with SiC, similar values of corrosion potential were obtained when the concentration of ceramic nanoparticles was 1 and 2 wt.%. When the SiC concentration increased to 3 and 4 wt.%, the corrosion potential values were above 100 mV more negative than reported in this work [41]. Slightly higher corrosion potential compared to this work, −740 mV vs. the Ag|AgCl (sat. KCl) reference electrode, was obtained by Cheng et al. for 7075 AAMC reinforced with SiC [39], and Murugabalaji et al. for hybrid composite reinforced with SiC and graphite [40]. However, more negative corrosion potentials, between −960 and −1160 mV vs. the Ag|AgCl (sat. KCl) reference electrode, were also reported [46].

#### 3.3.2. Corrosion Behaviour of Composite Materials in the Vicinity of the Corrosion Potential

**Figure 13:**
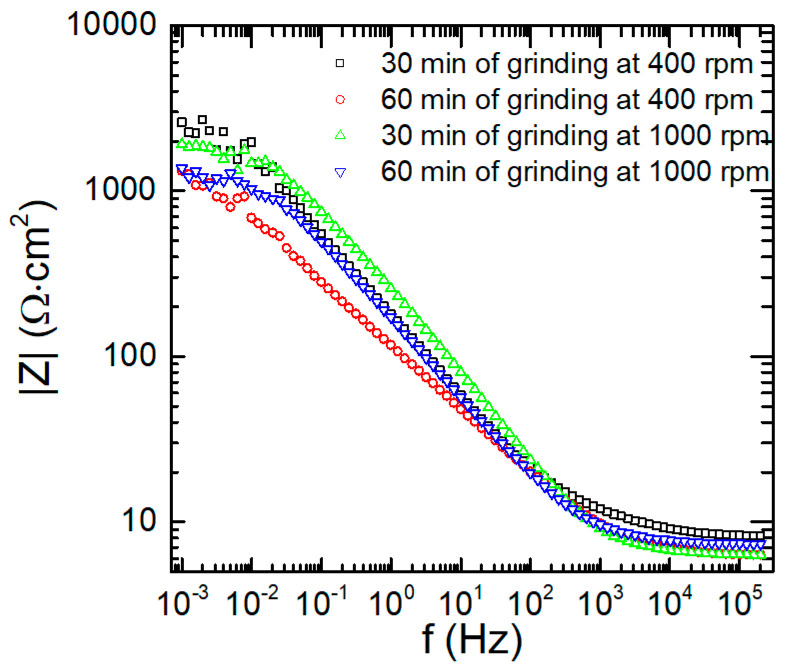
Bode magnitude plot for composite materials immersed in 3.5 wt.% NaCl solution, in equilibrium with air, *T* = 30 °C, as a function of their fabrication conditions i.e., grinding speed and total grinding time for the total concentration of ceramic particles 12 wt.%.

The corrosion susceptibility of the composite materials was studied using electrochemical impedance spectroscopy and cyclic voltammetry. Impedance spectra were obtained immediately after finishing the OCP measurements. Unfortunately, despite the relatively long period during which the specimens corroded freely, still the recorded values were scattered in the small mV range. This significantly deteriorated the quality of the impedance spectra at frequencies below 25 mHz. In addition, the corrosion mechanism itself is complex. Its analysis should include dissolution of the alloy, formation of the oxide layer, its destruction in the presence of chloride ions, and diffusion and reduction of possible depolarisers in the corrosion cell. The latter include oxygen dissolved in the electrolyte and small amounts of heavy metal ions such as Cu^2+^, Fe^2+^, and Zn^2+^. Heavy metal ions appear in the electrolyte from intermetallic particles. These particles may either directly corrode in contact with the matrix, because they are less noble, e.g., MgZn_2_ [38], or indirectly when they are first mechanically removed from the matrix. The latter occurs due to the corrosion of the matrix in the vicinity of the more noble intermetallic particle, such as Al_7_Cu_2_Fe and (Al,Cu)_6_(Fe,Cu), and was described, e.g., for 2024 aluminium alloy [59,60]. Intermetallic particles, when not in galvanic contact with aluminium, may undergo selective corrosion [61]. Thus, due to the high complexity of the studied system, we were unable to propose a reliable equivalent circuit and investigate the impedance spectra in detail. However, a simple measure of the relative corrosion resistance of the studied composite materials may be their impedance modulus at the lowest frequency used in the experiments, which should be inversely proportional to their corrosion rates. It can be observed in Figure 13 that there is no clear correlation between the manufacturing conditions of the composite materials and their corrosion resistance at the OCP. The lowest impedance modulus, of ca. 1.3 kΩ∙cm^2^, was obtained for the material ground at 400 rpm for 60 min. The highest impedance modulus, of ca. 2.6 kΩ∙cm^2^, was obtained for 400 rpm and 30 min of total grinding time. Higher values have been reported in the literature: up to 5 kΩ∙cm^2^ for 7075/SiC/graphite AAMC [40], and between 3 and 8 kΩ∙cm^2^ for 7075 alloy/short basalt fibre metal matrix composite [44], both at 0.1 Hz.

**Figure 14:**
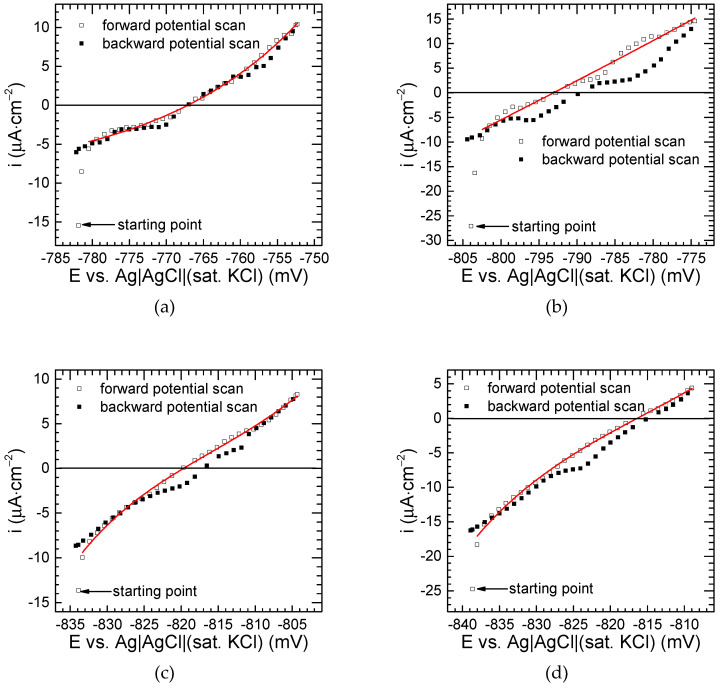
Voltammograms of composite materials immersed in 3.5 wt.% NaCl solution, in equilibrium with air, *T* = 30 °C, as a function of their fabrication conditions for the total concentration of ceramic particles 12 wt.%: (**a**) grinding for 30 min at 400 rpm; (**b**) grinding for 60 min at 400 rpm; (**c**) grinding for 30 min at 1000 rpm; (**d**) grinding for 60 min at 1000 rpm.

The corrosion rate and, to some extent, the corrosion mechanism, can be relatively easily obtained from voltammograms recorded in the vicinity of the corrosion potential (Figure 14). Composite materials investigated in this work were covered with the oxide layer during corrosion tests, because the pH of the corrosive environment, 3.5 wt.% NaCl solution, was close to neutral. This layer is spontaneously formed in the air, also during corrosion processes. It may be thickened during the polarisation of the specimen, when its potential increases (forward scans in Figure 14). This may cause slightly lower values of the current density recorded during the backward scan. In fact, the observed negative hysteresis on cyclic voltammograms recorded in a nearly neutral solution was rather small because the electrode had already been covered with the oxide before polarisation, but it can be more prominent in more aggressive corrosion environments [62].

The results obtained during the forward potential scans were approximated using Equation (2), as proposed by Mansfeld [63]:

(2) $$i=\frac{1}{R_{p}}\frac{b_{a}b_{c}}{b_{a}+b_{c}}\left[\exp\left(\frac{2.303\left(E-E_{\mathrm{corr}}\right)}{b_{a}}\right)-\exp\left(\frac{-2.303\left(E-E_{\mathrm{corr}}\right)}{b_{c}}\right)\right]$$

where *i* represents the current density, *E* is the potential, *E*_corr_ is the corrosion potential, *R*_p_ indicates polarisation resistance, and *b*_c_ and *b*_a_ represent the Tafel slopes for cathodic and anodic reactions, respectively. Approximation results are provided in Table 4. Coefficient of determination *R*^2^ values are close to unity, showing a relatively good fit. The corrosion potential is typically obtained from such an approximation with high precision. The obtained values correspond well to those from the OCP measurements. Only in the case of the composite material obtained during 60 min of grinding at 1000 rpm was the difference in *E*_corr_ equal to 9 mV. The polarisation resistances obtained for two specimens fabricated during 30 min of grinding were the highest and very similar to each other, which is consistent with their highest impedance modulus (Figure 13). The lowest *R*_p_ was obtained for the material fabricated during 60 min of grinding, at 400 rpm. Besides this value, all polarisation resistances obtained are slightly higher than the value of 1.5 kΩ∙cm^2^ reported for 7075 aluminium alloy in a T6 metallurgical state [58]. Because the corrosion resistance of composite materials can be related to both the properties of the matrix and the reinforcement, it should be noted that the matrix in our case is not in the T6 metallurgical state due to the sintering process; also, the corrosion tests were conducted by Rodiĉ and Miloŝev under less aggressive conditions, i.e., a lower chloride concentration, of approximately 0.6 wt.% and a lower temperature, of 25 °C.

Regarding the Tafel slopes, very high values of *b*_c_ were obtained for two composite materials fabricated at a low grinding speed. They approach the upper limit in the approximation algorithm, indicating that the cathodic process, that is, the reduction of dissolved oxygen and possibly small amounts of heavy metal ions, is diffusion-controlled [63]. The diffusion process is probably related to the porosity of the composite materials, because in the case of more compact specimens, manufactured at 1000 rpm, relatively low values of the cathodic Tafel slope, between 26 and 36 mV∙dec^−1^ were obtained. This, in turn, could be attributed to the influence of the oxide layer in the charge transfer process [62]. The same is true for *b*_a_ [62,64]. It remains unclear why such a high value of the anodic Tafel slope was obtained for the specimen fabricated during 60 min of grinding at 400 rpm. Tafel slopes for anodic and cathodic corrosion processes of 7075-based AAMCs in 3.5 wt.% of NaCl have already been reported in the literature. Bharathi obtained cathodic Tafel slopes in the range from 178 to 290 mV∙dec^−1^ and anodic slopes from 69 to 235 mV∙dec^−1^ with no correlation with the amount of added SiC [46]. Karunanithi et al. [48] extensively studied the influence of the amount of added TiO_2_ and the metallurgical state of the matrix, but the reported Tafel slopes are unusually high and change in a very wide range, from 253 to 1152 mV∙dec^−1^ with no apparent correlation with the conditions of composite fabrication. The Tafel slope values reported in both aforementioned publications are problematic. In the former, no details were provided about how they were obtained. In the latter case, the Tafel extrapolation method was applied, but the authors reported a problem with identification of the clearly defined linear parts of the polarisation curves. Therefore, they are difficult to discuss and compare with those reported in this work.

The corrosion rates of the AAMCs were calculated using Equation (3) [63]:

(3) $$i_{\mathrm{corr}}=\frac{1}{R_{p}}\frac{b_{a}b_{c}}{b_{a}+b_{c}}$$

The following results were obtained using data from Table 4: 9.58 μA∙cm^−2^ for 30 min of milling at 400 rpm, 341.47 μA∙cm^−2^ for 60 min of milling at 400 rpm, 3.24 μA∙cm^−2^ for 30 min of milling at 1000 rpm, and 4.58 μA∙cm^−2^ for 60 min of milling at 1000 rpm. However, these results are of limited practical importance and should not be converted to mm∙year^−1^ because the corrosion process has a non-uniform character, as is typical for aluminium alloys and aluminium alloy matrix composites. A very high value of the corrosion current density was obtained for the material fabricated during 60 min of grinding at 400 rpm due to the high values of Tafel constants and the low value of the polarisation resistance.

The corrosion behaviour of similar composite materials has already been studied. Suresh et al. determined the corrosion current density of 7075 alloy matrix composite reinforced with SiC nanoparticles. They found that the corrosion current density decreased from about 3.1 A∙cm^−2^ to 2.5 A∙cm^−2^ as the SiC concentration increased from 1 to 4 wt.%. These values are around 10^6^ times higher compared to those reported in this work. Unfortunately, no details on how these values were obtained were provided [41]. Murugabalaji et al. investigated the effect of thermomechanical processing on the corrosion resistance of a 7075 aluminium alloy composite containing 2 wt.% of SiC and 0.5 wt.% of graphite. The corrosion current densities obtained were in the range between 8.8 and 45.6 μA∙cm^−2^ depending on the thermomechanical processing applied. These values are also higher than those obtained in our work. This may be due to the presence of graphite and its electrochemical coupling with the matrix, but this was not discussed by the authors [40]. Over ten-fold lower corrosion current density compared to our work, i.e., 0.324 μA∙cm^−2^, was obtained for the 7075 alloy matrix composite material containing 12 wt.% of SiC [46]. The corrosion properties of the TiO_2_-reinforced 7075 AAMC were also extensively studied [48]. When the reinforcement was in the “as received” state, the corrosion current density of the composite containing 5 vol.% of reinforcement was 111 μA∙cm^−2^ for the T4 metallurgical state. Artificial aging of the obtained composite was also carried out and the highest *i*_corr_ = 298 μA∙cm^−2^ was obtained for the peak-aged material (24 h at 110 °C). In general, reported values are significantly higher compared to the values obtained in this work, with the only exception being the material ground for 60 min at 400 rpm. When the TiO_2_ powder was ground for 20 h prior to composite fabrication, different results were obtained. The highest corrosion rate was obtained for the T4 metallurgical state, of 412 μA∙cm^−2^. For the under-aged matrix condition, 102 μA∙cm^−2^ was obtained. For the peak-aged and over-aged materials, corrosion current densities were considerably lower, at 8.8 and 5.3 μA∙cm^−2^, respectively.

It should be noted that all of the aforementioned corrosion current densities were obtained using the Tafel extrapolation method. With a lack of detailed information on how this procedure was conducted and without clearly defined Tafelian regions on the polarisation curves, the results reported are difficult to compare with those obtained in our work.

The corrosion behaviour of the hybrid composite material containing SiC and TiO_2_ as reinforcement with the matrix made of the A356 aluminium cast alloy was studied in 2 wt.% NaCl solution. The corrosion rate of the material containing 5 wt.% of SiC and 0.5 wt.% of TiO_2_ was equal to approximately. 23.8 mA∙cm^−2^. This value was calculated from the results of the weight loss test after 24 h of immersion, i.e., it is the average value of the corrosion rate. However, considering that the OCP of composite materials in our work was fairly stable with time, the reported corrosion current density could be comparable. Thus, the difference of more than one order of magnitude is surprising even despite the different matrix [65].

**Figure 15:**
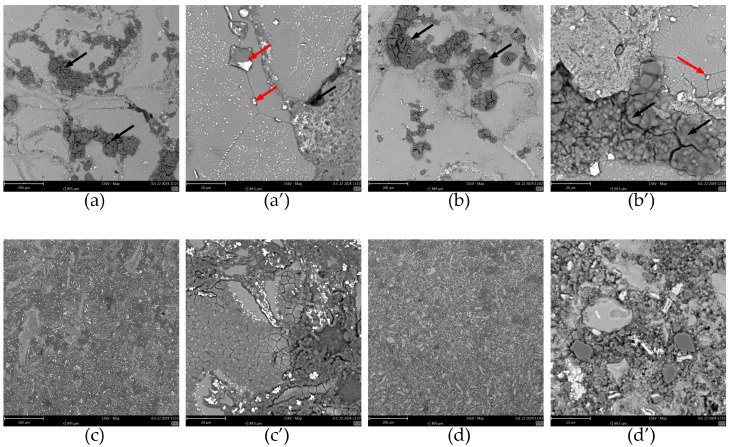
SEM micrographs of the surface of the composite materials immersed in 3.5 wt.% NaCl solution, in equilibrium with air, *T* = 30 °C, *t* = 24 h as a function of their fabrication conditions for the total concentration of ceramic particles 12 wt.%: (**a**) grinding for 30 min at 400 rpm; (**a’**) grinding for 30 min at 400 rpm, magnified areas showing matrix grains and intermetallic particles; (**b**) grinding for 60 min at 400 rpm; (**b’**) grinding for 60 min at 400 rpm, magnified areas showing matrix grains and intermetallic particles; (**c**) grinding for 30 min at 1000 rpm; (**c’**) grinding for 30 min at 1000 rpm, magnified areas showing matrix grains and intermetallic particles; (**d**) grinding for 60 min at 1000 rpm; (**d’**) grinding for 60 min at 1000 rpm, magnified areas showing matrix grains and intermetallic particles;. Arrows indicate corrosion attack.

When cyclic voltammograms in the vicinity of the corrosion potential were recorded, specimens were removed from the solution and rinsed with demineralised water, and their surface was examined using SEM (Figure 15). In the case of materials obtained at a 400 rpm grinding speed (Figure 15a,a’,b,b’), the formation of an insoluble product of corrosion of aluminium was detected at the interphase between the matrix and the reinforcement. These areas are indicated with black arrows in Figure 15. Such an extensive formation of the corrosion product was not observed on the surface of the specimens obtained at a 1000 rpm grinding speed (Figure 15c,c’,d,d’). Therefore, the quality of contact between the aluminium alloy chips and the reinforcement determines the corrosion resistance of the composite [1]. When it is poor, there are crevices where the electrolyte could penetrate. If the access of dissolved oxygen is restricted, aluminium dissolution should occur within these crevices. This may cause an increase in the acidity of the solution within the crevices and an enhanced dissolution of the aluminium. However, the corrosion process is not restricted to the metal/reinforcement interphase. In the case of materials obtained from powders ground at 400 rpm, where the aluminium alloy particles were sufficiently large, corrosion occurred around the intermetallic phases. This is indicated with red arrows in Figure 15a’,b’. These particles containing copper or copper and iron are more noble than the matrix and induce its corrosion. This mechanism is the same as in the case of 7075 aluminium alloy [38]. The grain boundaries within these relatively large aluminium alloy particles were also exposed. Thus, the corrosion properties of the composite materials obtained can be controlled on two levels. The first is to ensure a good connection between the matrix and the reinforcement. The second is the heat treatment of the composite. This affects the size and distribution of the intermetallic particles and thus corrosion within the aluminium alloy grains.

#### 3.3.3. Anodic Polarisation of the Composite Materials

Anodic polarisation curves are typically recorded for materials with a high ability to form a passive layer. From these curves, the protective quality of the oxide layer formed on the surface of aluminium alloys and AAMCs can be estimated. Two features of the anodic polarisation curves will be analysed. The first is the value of current density during polarisation. If this is low, the rate of metal dissolution is also low, which means good protective properties of the passive layer. When the metal is anodically polarised in the solution containing, e.g., chloride ions, at a certain potential a local destruction of the passive layer occurs and the rapid increase in the current density is observed. This potential is called the breakdown potential, and its values will also be determined in this work. In this case, the higher the value of the breakdown potential, the better the protective properties of the passive layer. It is important to note that from the polarisation curves it cannot be determined what mechanism is responsible for the breakdown of the passive layer, and additional studies are necessary.

**Figure 16:**
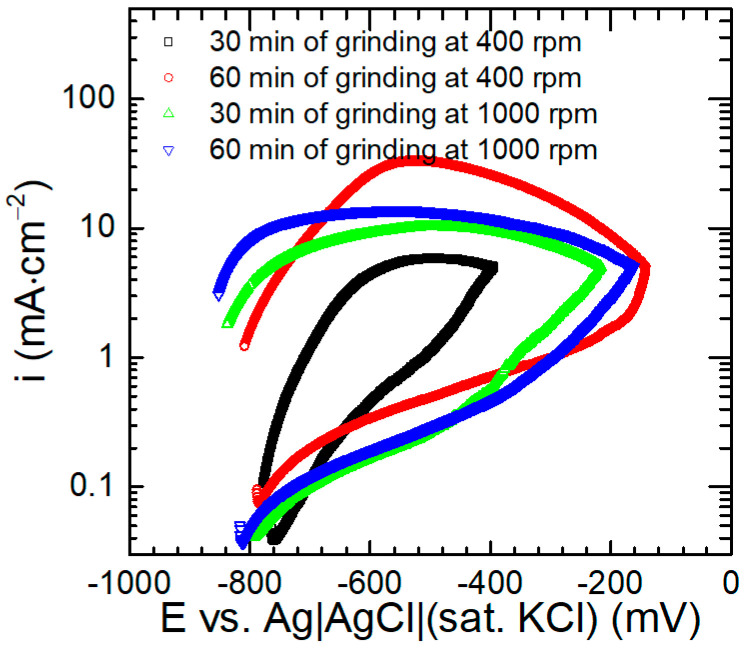
Anodic polarization curves of the composite materials immersed in 3.5 wt.% NaCl solution, in equilibrium with air, *T* = 30 °C, as a function of their fabrication conditions i.e., grinding speed and total grinding time for the total concentration of ceramic particles 12 wt.%.

In this work, instead of single polarisation curves, the cyclic voltammograms were measured with the working electrodes polarised anodically from the OCP to a certain vertex potential and then back to the OCP (Figure 16). First, it can be concluded that there are no clearly defined passive regions on the recorded polarisation curves. Instead, a constant increase in the current density with increasing potential was observed. This increase in current density was the steepest for the composite material obtained during 30 min of grinding, at 400 rpm. In the case of the composite material obtained during 60 min of grinding at 400 rpm, recorded current densities were initially higher, but their increase with increasing potential was less steep, and at sufficiently positive potential, lower current densities were obtained. Current densities recorded during polarisation of the two composite material obtained at a grinding rate of 1000 rpm were very similar to each other and lower compared to the materials obtained at 400 rpm. This suggests better corrosion resistance of the materials obtained at the high grinding rate, in accordance with the results obtained at the OCP. Unfortunately, the interpretation of the obtained results becomes more complicated when we take into account the breakdown potential values. There was no clear breakdown potential for the material ground for 30 min at 400 rpm, but when the grinding time was doubled, to 60 min, the highest value of the breakdown potential, of ca. −175 mV vs. the Ag|AgCl (sat. KCl) reference electrode, was obtained. This potential for materials obtained during 30 and 60 min of grinding, at 1000 rpm, was approximately −330 and −430 mV, respectively. Lower breakdown potentials, around −760 mV vs. the Ag|AgCl (sat. KCl) reference electrode, were reported in the literature, suggesting a higher susceptibility of the composite materials to the localised corrosion processes. In this work, no significant influence of SiC or B_4_C on the breakdown potential was observed [46]. In the case of 7075/short basalt fibre metal matrix composite, no clear breakdown potential was observed, but anodic current densities were much higher compared to this work. For instance, for ca. −400 mV anodic overpotential, *i* = 30 mA∙cm^−2^ [44] was obtained, compared to around 1 mA∙cm^−2^ in this work (Figure 16).

Corrosion during anodic polarisation of all the specimens was related both to matrix dissolution around intermetallic particles and in the vicinity of the reinforcing particles, because these two processes already occurred at the OCP (Figure 15). When the current density of 5 mA∙cm^−2^ was achieved, the polarisation direction changed. Because the conditions for further aluminium dissolution became more favourable than initially due to anodic polarisation, for instance due to pH decrease within the crevices, current densities during the backward scan were significantly higher compared to those during the forward scan. These high current density values were responsible for most of the corrosion damage observed on the specimens.

**Figure 17:**
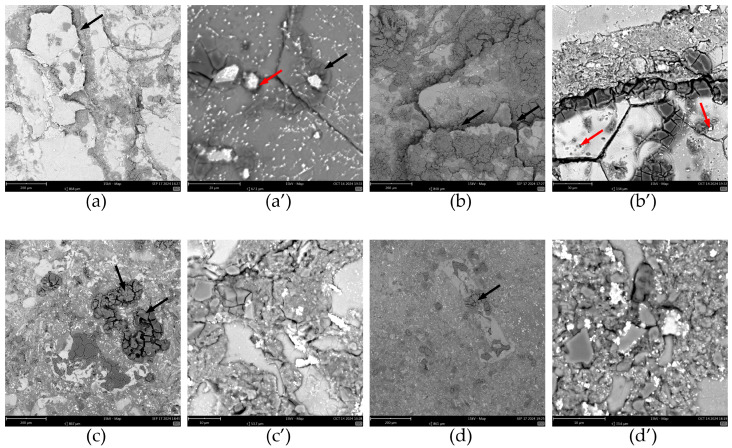
SEM micrographs of the surface of the composite materials immersed in 3.5 wt.% NaCl solution, in equilibrium with air, *T* = 30 °C, after anodic polarisation, as a function of their fabrication conditions for the total concentration of ceramic particles 12 wt.%: (**a**) grinding for 30 min at 400 rpm; (**a’**) grinding for 30 min at 400 rpm, magnified areas showing matrix grains and intermetallic particles; (**b**) grinding for 60 min at 400 rpm; (**a’**) grinding for 60 min at 400 rpm, magnified areas showing matrix grains and intermetallic particles; (**c**) grinding for 30 min at 1000 rpm; (**c’**) grinding for 30 min at 1000 rpm, magnified areas showing matrix grains and intermetallic particles; (**d**) grinding for 60 min at 1000 rpm; (**d’**) grinding for 60 min at 1000 rpm, magnified areas showing matrix grains and intermetallic particles. Arrows indicate corrosion attack..

Interpretation of the breakdown potential required SEM analysis of the specimens after registering anodic polarisation curves. It can be concluded that the rapid increase in the anodic reaction rate was not related to the pitting corrosion, as no corrosion pits were found, but rather to intense metal dissolution within the crevices at the matrix/reinforcement interphase (Figure 17). In the case of composite materials manufactured at 400 rpm, a significant amount of corrosion product formed within the crevices (black arrows in Figure 17a,b). Because of the layered microstructure of these materials, platelet-like structures were dislodged, and the effect analogous to exfoliation corrosion was observed. The corrosion of the matrix around the intermetallic particles and along the grain boundaries also occurred (red arrows in Figure 17a’,b’). In the case of composites obtained during grinding at 1000 rpm, only a moderate corrosion attack occurred at the matrix/reinforcement interphase (Figure 17c,d). It is interesting that there was no corrosion around the intermetallic particles within the matrix (Figure 17 c’,d’). This was observed only in the case of relatively large aluminium alloy particles. This means that the grinding rate not only improved the corrosion resistance of the composites by improving the adhesion at the matrix/reinforcement interphase, but also mitigated corrosion within the alloy grains.

## 4. Conclusions

First, it was shown that high-energy ball milling can be used successfully to obtain compact composite material from 7075 aluminium alloy machining chips with a relatively short grinding time. Importantly, the chips did not require extensive chemical pretreatment. Such a pretreatment can involve degreasing and simultaneous etching in an alkaline solution to remove the remaining machining coolant and the oxide layer. Al_2_O_3_ dissolves easily in alkaline solution, but this process can be difficult to control due to the very high surface area of the chips. When the oxide layer is removed, corrosion of the aluminium alloy occurs and hydrogen is generated. The dissolution of aluminium and aluminium oxide is an exothermic process, and increasing temperature further accelerates the corrosion of aluminium and hydrogen evolution. Thus, in the case of materials with a high surface-to-volume ratio, such as machining chips, a significant amount of hydrogen can be generated, which is potentially dangerous. Furthermore, extensive etching changes the chemical composition of the surface of the chips. They can become significantly enriched with alloying elements that are more noble compared to aluminium, e.g., copper. This may also complicate further processing. An interesting question is what actually happens with the aluminium oxide during grinding. It was deduced from the microscopic examinations of the composite materials obtained that aluminium oxide was probably removed from the surface of the chips during grinding and may constitute an additional reinforcement if it is homogeneously distributed between the matrix grains. This process can be better understood in the aluminium alloy–SiC system, without the oxide added as the reinforcement.

Second, the influence of the high-energy ball milling parameters, i.e., milling time and speed, as well as the amount of reinforcement added, on the microstructure of the obtained composite materials, was studied. It was found that the average particle size necessary to obtain homogeneous and nonporous material was around 28–36 μm. This was achieved during 60 min of milling at a 1000 rpm rotational speed. In general, the grinding speed, among the grinding parameters studied, had the strongest influence on the size distribution of the obtained powders. Ceramic particles also facilitate the fragmentation of aluminium chips. This effect was visible at a lower grinding speed and grinding time. From a practical point of view, the proper microstructure of the composite should be obtainable in a relatively large range of reinforcement concentration, with the latter responsible for, e.g., mechanical properties of the material. When the chips were ground for 60 min at 1000 rpm, the homogeneous and relatively nonporous composite material was obtained regardless of the quantity of ceramic particles added.

Third, the results of the corrosion studies underlined the role of the microstructure of the composite. The corrosion process occurred mainly at the matrix/reinforcement interphase. In the case of composites with relatively high porosity at this interphase and a layered microstructure, a sufficiently large amount of the corrosion product can be generated to endanger the structural integrity of the material. It was also observed that corrosion within the aluminium alloy grains, around the intermetallic particles that are more noble than the matrix, occurred only when the aluminium alloy grains were sufficiently large. In other words, the high grinding speed helped avoiding corrosion within the aluminium alloy grains.

Aluminium alloy matrix composites are generally applied in the aerospace industry, automotive industry, and construction, with the composites reinforced with ceramic particles mainly used for the fabrication of pistons, engine blocks, and brakes, due to their high wear resistance. Other possible applications that may involve contact with a corrosive environment are components of hydraulic systems, e.g., hydraulic gears and valves, marine ship body parts, or components of aircraft undercarriage legs. The more specific future application of the obtained material would be indicated after a thorough characterisation of its mechanical properties. At this moment, the practical importance of this study lies mainly in the selection of the grinding parameters necessary for the fabrication of the composite materials.

## Figures and Tables

**Table 1:** Nominal chemical composition of 7075 alloy, in wt.% [52].

Cu	Mn	Mg	Zn	Si	Fe	Ti	Al
1.2–2.0	<0.3	2.1–2.9	5.1–6.1	<0.4	<0.5	<0.1	bal.

**Table 2:** Composition of milled materials and milling parameters.

Milled Material Composition (wt.%)	Rotational Speed (rpm)	Total Grinding Time (min)
97% 7075 alloy 1.5% SiC 1.5% TiO_2_	400	30
		60
	1000	30
		60
94% 7075 alloy 3% SiC 3% TiO_2_	400	30
		60
	1000	30
		60
88% 7075 alloy 6% SiC 6% TiO_2_	400	30
		60
	1000	30
		60

**Table 3:** Porosity of the composite materials.

Grinding Conditions	Volume Fraction of Porosity (%)	Standard Deviation	Analysed Area (µm^2^)
400 rpm/30 min	5.31	3.15	72,352.03
400 rpm/60 min	4.70	0.84	
1000 rpm/30 min	0.86	0.23	11,571.27
1000 rpm/60 min	0.70	0.20	

**Table 4:** The results of the approximation of the polarisation curves from Figure 14 using Equation (2).

Grinding Conditions	*R* ^2^	*E*_corr_ (mV) ^1^	*R*_p_ (kΩ∙cm^2^)	*b*_a_ (mV∙dec^−1^)	*b*_c_ (mV∙dec^−1^)
30 min, 400 rpm	0.9952	−767	2.084 ± 0.042	46 ± 4	9.5 × 10^18 2^
60 min, 400 rpm	0.9861	−793	1.250 ± 0.050	983 ± 2667	2.8 × 10^18 2^
30 min, 1000 rpm	0.9976	−820	2.001 ± 0.055	35 ± 4	26 ± 2
60 min, 1000 rpm	0.9972	−816	1.751 ± 0.041	38 ± 10	36 ± 4

^1^ Corrosion potential is provided vs. the Ag|AgCl (sat. KCl) reference electrode; uncertainties obtained from the fit are <1 mV. ^2^ These values approach the upper limit of cathodic Tafel constant in the approximation algorithm, 10^100^, and can be simply regarded as infinite, i.e., the cathodic reaction is under diffusional control.

## Data Availability

The original data presented in the study are openly available in a RepOD Open Repository at https://doi.org/10.18150/9RT09P.
